# The coral reef-dwelling *Peneroplis* spp. shows calcification recovery to ocean acidification conditions

**DOI:** 10.1038/s41598-022-10375-w

**Published:** 2022-04-16

**Authors:** Laurie M. Charrieau, Yukiko Nagai, Katsunori Kimoto, Delphine Dissard, Beatrice Below, Kazuhiko Fujita, Takashi Toyofuku

**Affiliations:** 1 Institute for Extra-Cutting-Edge Science and Technology Avant-Garde Research (X-STAR), Japan Agency for Marine-Earth Science and Technology (JAMSTEC), Natsushima-cho 2-15, Yokosuka, 237-0061 Japan; 2 Marine Biogeosciences, Alfred Wegener Institute (AWI), Am Handelshafen 12, 27570 Bremerhaven, Germany; 3 National Museum of Nature and Science, 4-1-1 Amakubo, Tsukuba, 305-0005 Japan; 4 Research Institute for Global Change (RIGC), Japan Agency for Marine-Earth Science and Technology (JAMSTEC), Natsushima-cho 2-15, Yokosuka, 237-0061 Japan; 5 IRD/UMR LOCEAN (IRD-CNRS-MNHN-Sorbonne Université), Centre IRD de Nouméa, 101 Promenade Roger Laroque, 98848 Nouméa, New Caledonia; 6 CR2P/UMR 7207 (Sorbonne Université-CNRS-MNHN), 4 Place Jussieu, 75005 Paris, France; 7 Department of Physics and Earth Sciences, University of the Ryukyus, Okinawa, Japan; 8 Tokyo University of Marine Science and Technology (TUMSAT), Konan 4-5-7, Minato, Tokyo, 108-8477 Japan

**Keywords:** Marine biology, Climate-change impacts, Biogeochemistry

## Abstract

Large Benthic Foraminifera are a crucial component of coral-reef ecosystems, which are currently threatened by ocean acidification. We conducted culture experiments to evaluate the impact of low pH on survival and test dissolution of the symbiont-bearing species *Peneroplis* spp., and to observe potential calcification recovery when specimens are placed back under reference pH value (7.9). We found that *Peneroplis* spp. displayed living activity up to 3 days at pH 6.9 (Ω_cal_ < 1) or up to 1 month at pH 7.4 (Ω_cal_ > 1), despite the dark and unfed conditions. Dissolution features were observed under low Ω_cal_ values, such as changes in test density, peeled extrados layers, and decalcified tests with exposed organic linings. A new calcification phase started when specimens were placed back at reference pH. This calcification’s resumption was an addition of new chambers without reparation of the dissolved parts, which is consistent with the porcelaneous calcification pathway of *Peneroplis* spp. The most decalcified specimens displayed a strong survival response by adding up to 8 new chambers, and the contribution of food supply in this process was highlighted. These results suggest that porcelaneous LBF species have some recovery abilities to short exposure (e.g., 3 days to 1 month) to acidified conditions. However, the geochemical signature of trace elements in the new calcite was impacted, and the majority of the new chambers were distorted and resulted in abnormal tests, which might hinder the specimens’ reproduction and thus their survival on the long term.

## Introduction

Anthropogenic carbon dioxide (CO_2_) emissions in the atmosphere are consistently increasing, which is driving the current climate change including the ocean acidification (OA) phenomenon^1–3^. The majority of calcifying marine organisms are negatively affected by OA and display decreased survival, calcification rate, growth and abundance when living under OA conditions^4,5^. However, some species appear not to be affected or even benefit from OA conditions, and thus the response of calcifiers to OA is complex and depends on several parameters such as their life stage, nutritional status, protective organic covering, pH regulation and photosynthesis abilities^4–6^. Moreover, local adaptation and adaptive plasticity appear to have a role in the species resilience to OA^7^. Coral reefs were among the first ecosystems to be recognized as threatened by OA^8^, and models predict heterogeneous but rapid degradation of coral reefs worldwide under future climate change scenarios^9–11^.

Large Benthic Foraminifera (LBF) are calcifying protists dwelling in warm coral reef environments, where they contribute to almost 5% of the annual present day calcium carbonate production^12,13^. Most modern LBF are hosts of algal symbionts, which provide them a valuable source of energy through photosynthesis^14,15^. This photosynthetic activity locally increases pH around the foraminifera, which partially protect them but does not fully compensate for OA conditions^16,17^. Foraminifera are also known to have a tight control of their internal and external pH during the calcification process^18–20^. Benthic foraminifera are widely used in fossil and modern records as biostratigraphic markers and bioindicators, including in coral-reef environments^21–23^. Furthermore, the trace elements ratios to calcium in their tests usually reflects the physical and chemical conditions in the calcification environment, providing useful tools for tracking of past and present environmental changes^24^. Most LBF species are made of high-Mg calcite, a mineral more prone to dissolution than low-Mg calcite under low pH values^25,26^, and thus their elemental composition has become of increasing interest in the OA context. However, only few recent studies reported on LBF multi-elemental signatures, and contrasted effects of OA on elemental ratios (E/Ca) were observed depending on the species and on the studied carbonate system parameter^27–29^.

The calcification response to OA conditions is not uniform among LBF species, and notably depends on: (1) the type of symbionts they host, (2) their nutritional dependency to them, and (3) their calcification pathways (hyaline or porcelaneous forms)^30^. In culture experiments, the calcification rates of some hyaline species hosting diatoms were mostly unaffected and even increased under low pH conditions^31–34^, whereas porcelaneous species hosting dinoflagellates or chlorophytes tend to have reduced calcification rates under low pH^31,32,35,36^, but opposite responses were also observed^33,37^. Furthermore, negative synergistic and/or additive effects on the calcification process of LBF maintained in culture experiments were reported when OA conditions were combined with eutrophication^38^, warming^39–42^, or local stressor such as copper exposure^43^. These negative trends were confirmed by field studies implemented near naturally low pH environments such as around CO_2_ seeps or groundwater springs, where abundance of LBF were reduced and the assemblages shifted toward non-calcifying species^42,44–47^. In several studies involving diverse calcifying species including SBF (Small Benthic Foraminifera), access to a sufficient food source was suggested as a crucial condition in order to cope with OA conditions^6,48,49^.

While many studies have focused on low pH effects on LBF survival and calcification^50^, fewer have investigated their potential recovery when they are subsequently put back in culture under in situ average seawater pH values. Re-calcification features after decalcification events were observed in few cases on low-Mg calcite SBF from temperate environments in both culture experiments (*Ammonia beccari*^51^; *Rosalina leei*^52^), and on the field (*Ammonia aomoriensis*^53^).

This study focuses on *Peneroplis* spp., a porcelaneous high-Mg calcite LBF species found free-living in tropical shallow-water environments. They host rhodophytes symbionts, giving them a light purple color^15,54^. Their tests have non-lamellar walls, and an outer mineralized surface (extrados) made of aligned rod-shaped crystals^55^. Specimens of *Peneroplis* spp. were exposed to various low pH conditions during short (3 days) and longer (1 month) culture experiments, and in dark conditions (periods called the decalcification phases). We observed the recovery of the specimens once put back under average seawater pH conditions and day/night cycles for several weeks (periods called the recovery phases), through survival and calcification’s resumption abilities. In addition, two feeding treatments were tested. Decalcification and calcification’s recovery features of the specimens were documented in terms of external aspect, ultrastructure, and test density, while trace element concentrations of the specimens displaying newly precipitated calcite were analyzed.

## Results

### Decalcification phases

**Figure 1:**
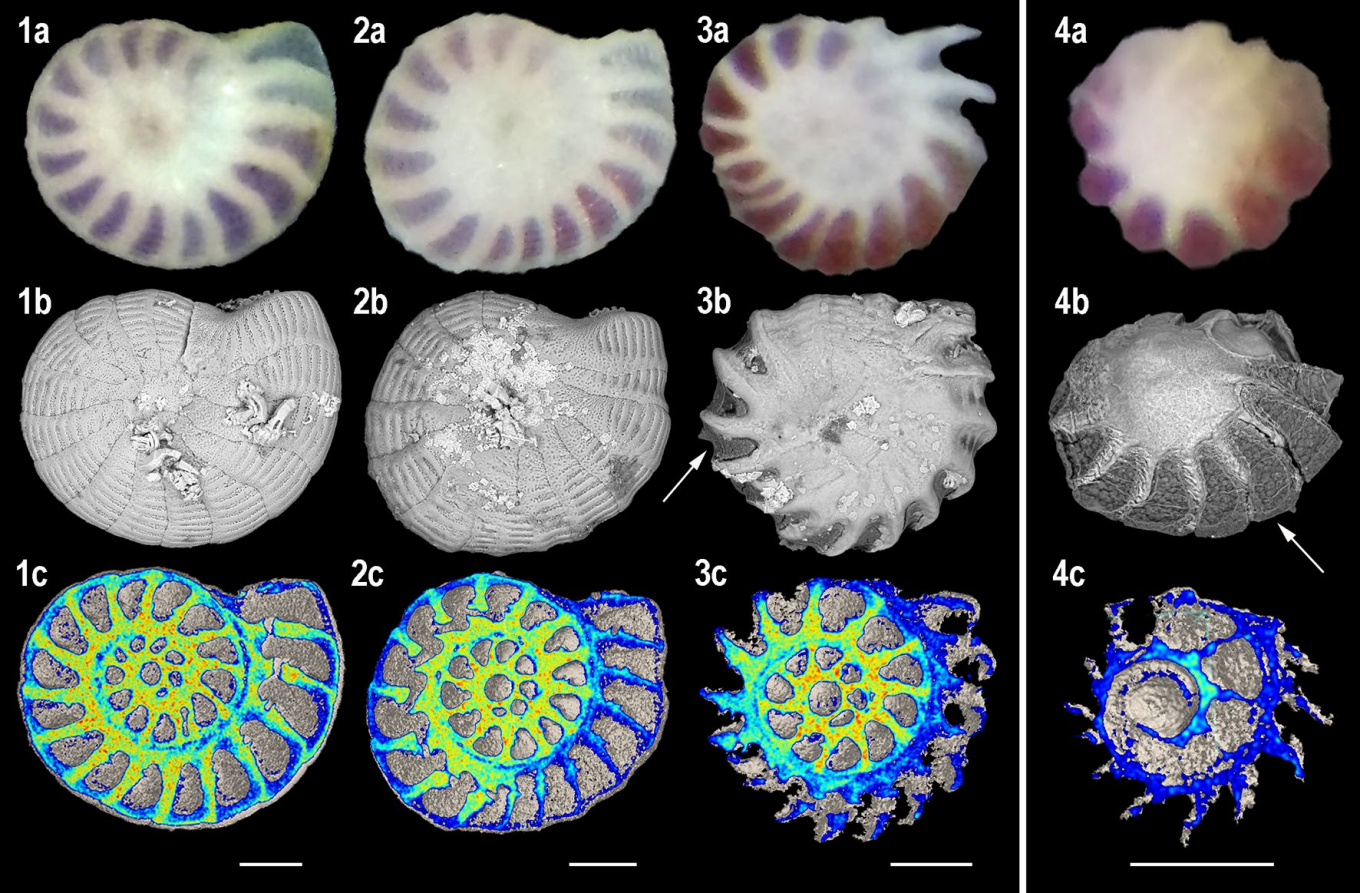
Light micrographs (above), SEM micrographs (middle) and cross-sections (below) of *Peneroplis* spp. specimens after the decalcification phases. Three days at (**1**) pH 7.9; (**2**) pH 7.4; (**3**) pH 6.9; (**4**) 32 days at pH 7.4. Colors are relative densities. Scale bars are 100 µm. Arrows: organic lining.

**Figure 2:**
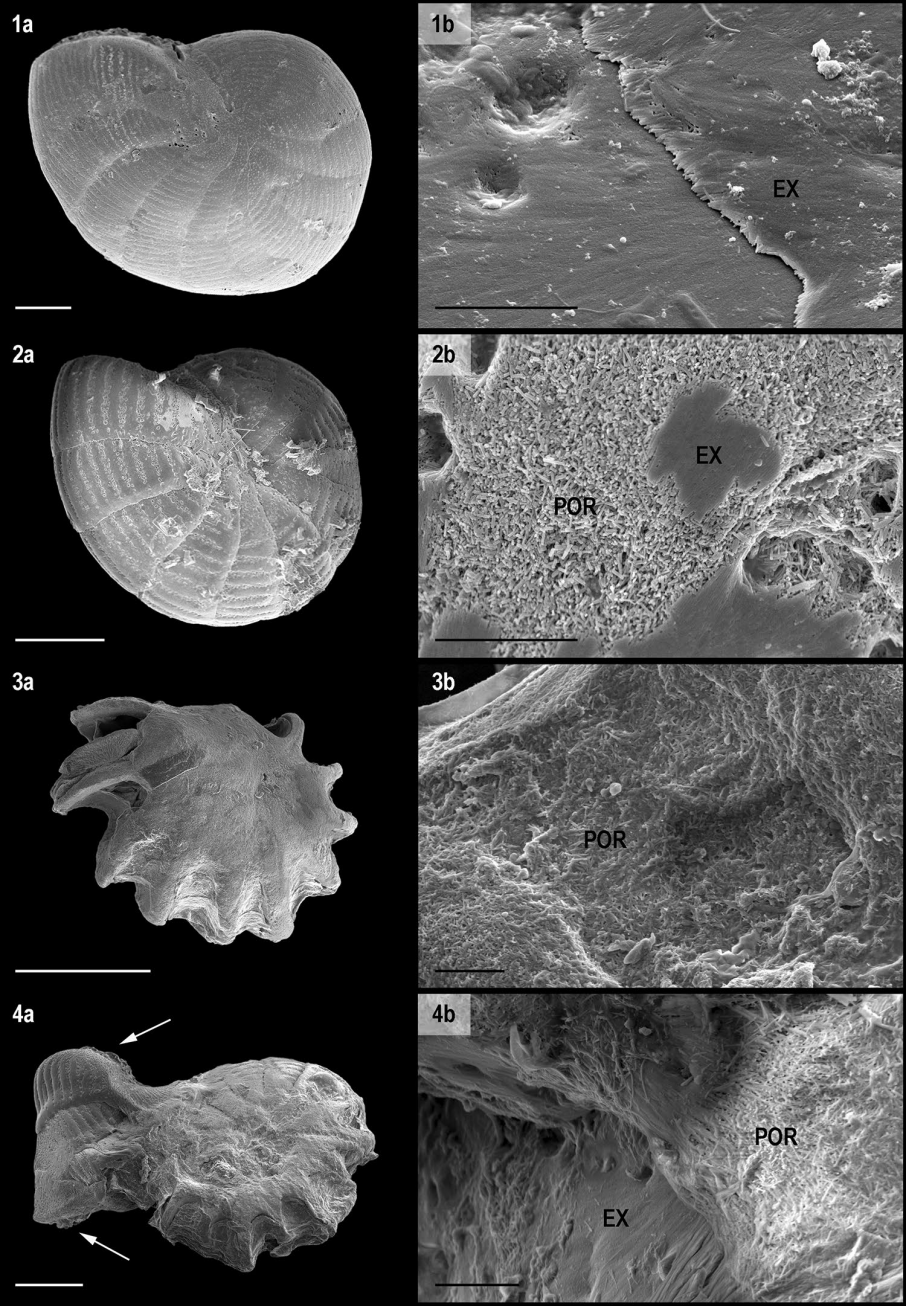
E-SEM micrographs of *Peneroplis* spp. Left: general views, scale bars are 100 µm. Right: magnified views, scale bars are 5 µm. Three days at (**1**) pH 7.9; (**2**) pH 7.4; (**3**) pH 6.9. (**4**) 32 days at pH 7.4 + 15 days at pH 7.9. *POR* porcelain, *EX* extrados. Arrows: apertures.

At the end of the decalcification phases, all the specimens displayed cytoplasm streaming and/or pseudopodia emission, and were thus considered alive. Even so, none of the specimens added new chambers during the decalcification phases. In experiment 1, after 3 days under pH 7.9 (reference conditions) or 7.4 (Ω_cal_ > 1; Table 1), test dissolution features on the light and SEM micrographs were unclear (Fig. 1: 1, 2). However, decalcification zones were distinctly visible on the high magnification E-SEM micrographs of one of the specimens kept under pH 7.4 (Fig. 2: 2a). On these zones, the extrados layer was peeled and the randomly oriented needles-shaped crystals of the under porcelain appeared (Fig. 2: 2b). After 3 days under pH 6.9 (Ω_cal_ < 1; Table 1), all the specimens displayed strongly decalcified tests on the light and SEM micrographs, and the inner organic lining protecting the cytoplasm and rhodophyte symbionts was largely exposed (Fig. 1: 3a, 3b). On the residual test observed by E-SEM imaging, the extrados layer was absent, and only some partly dissolved crystals of the porcelain were visible on the test surface (Fig. 2: 3b). In experiment 2, after 32 days under pH 7.4 (Ω_cal_ > 1; Table 1), the specimens presented the most decalcified features from both experiments, and the remaining calcite was only observed near the central part and chambers’ sutures of the tests (Fig. 1: 4a, b, c). The living symbionts were still clearly visible through the organic lining.

**Table 1:** Water chemical variables in each tank for the two experiments. Temperature, salinity, pH and total alkalinity were measured at the beginning, middle and end of the experiment; Ω_cal_, [CO_3_^2−^] and [Ca^2+^] were calculated.

		T (°C)	Average salinity (‰)	Average pH (pH unit)	Total alkalinity at 25 °C (μeq.kg^−1^)	Ω_cal_	[CO_3_^2−^] (μmol .kg.sw^−1^)	[Ca^2+^] (mmol.kg.sw^−1^)
Exp. 1	Tank 1	25	36.00 ± 0.00	7.94 ± 0.01	2537.86 ± 53.74	4.73	198.31	10.58
	Tank 2	25	34.33 ± 0.47	7.44 ± 0.00	2383.84 ± 10.54	1.58	65.27	10.09
	Tank 3	25	35.00 ± 0.00	6.93 ± 0.01	2458.35 ± 23.76	0.53	22.14	10.29
Exp. 2	Tank 1	25	37.33 ± 1.70	7.91 ± 0.01	2582.81	4.61	195.27	10.97
	Tank 2	25	34.00 ± 0.00	7.46 ± 0.03	2407.44 ± 7.96	1.66	68.33	9.99

**Figure 3:**
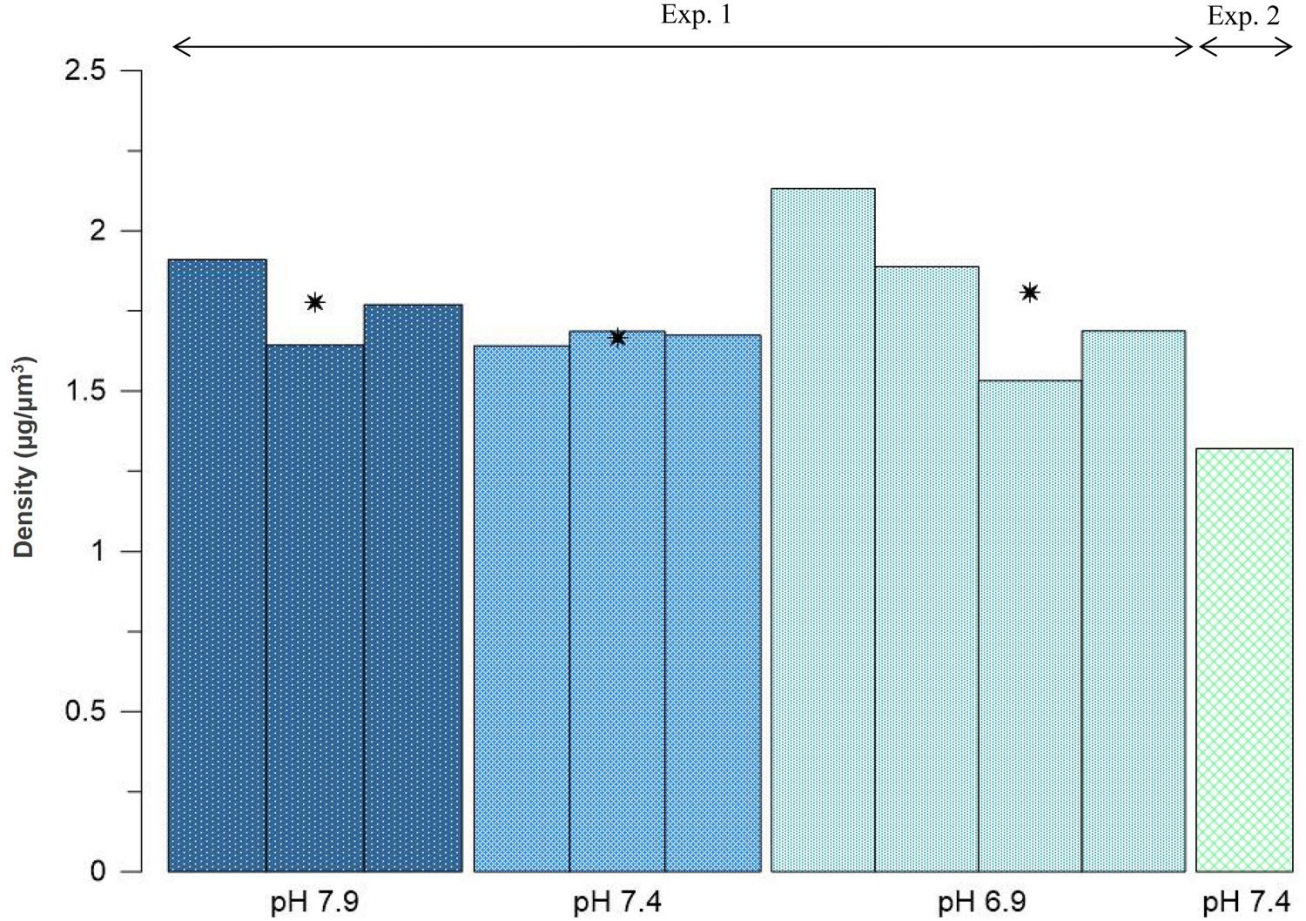
Test density of *Peneroplis* spp. specimens after 3 days at pH 7.9, 7.4 and 6.9 (Exp. 1), and after 32 days at pH 7.4 (Exp. 2). Each bar corresponds to one specimen. Stars (*) show the mean value for each pH condition.

In experiment 1, test densities had on average a higher value after the 7.9 pH reference treatment compared to after the 7.4 pH treatment (1.77 and 1.67 μg/μm^3^, respectively; Fig. 3), which suggests loss of calcite. The average test density was the highest after the 6.9 treatment (1.81 μg/μm^3^). The softest parts of test being completely dissolved and therefore absent (Fig. 1: 3a, b, c), the calcite left was probably the hardest and densest, leading to the overall higher densities of these specimens. At the end of experiment 2, the test density of one specimen was the lowest observed from both experiments (1.32 μg/μm^3^; Fig. 3). In that case, intense calcite loss likely happened from the whole test, including from the hardest parts (Fig. 1: 4a, b, c).

### Recovery phases

**Figure 4:**
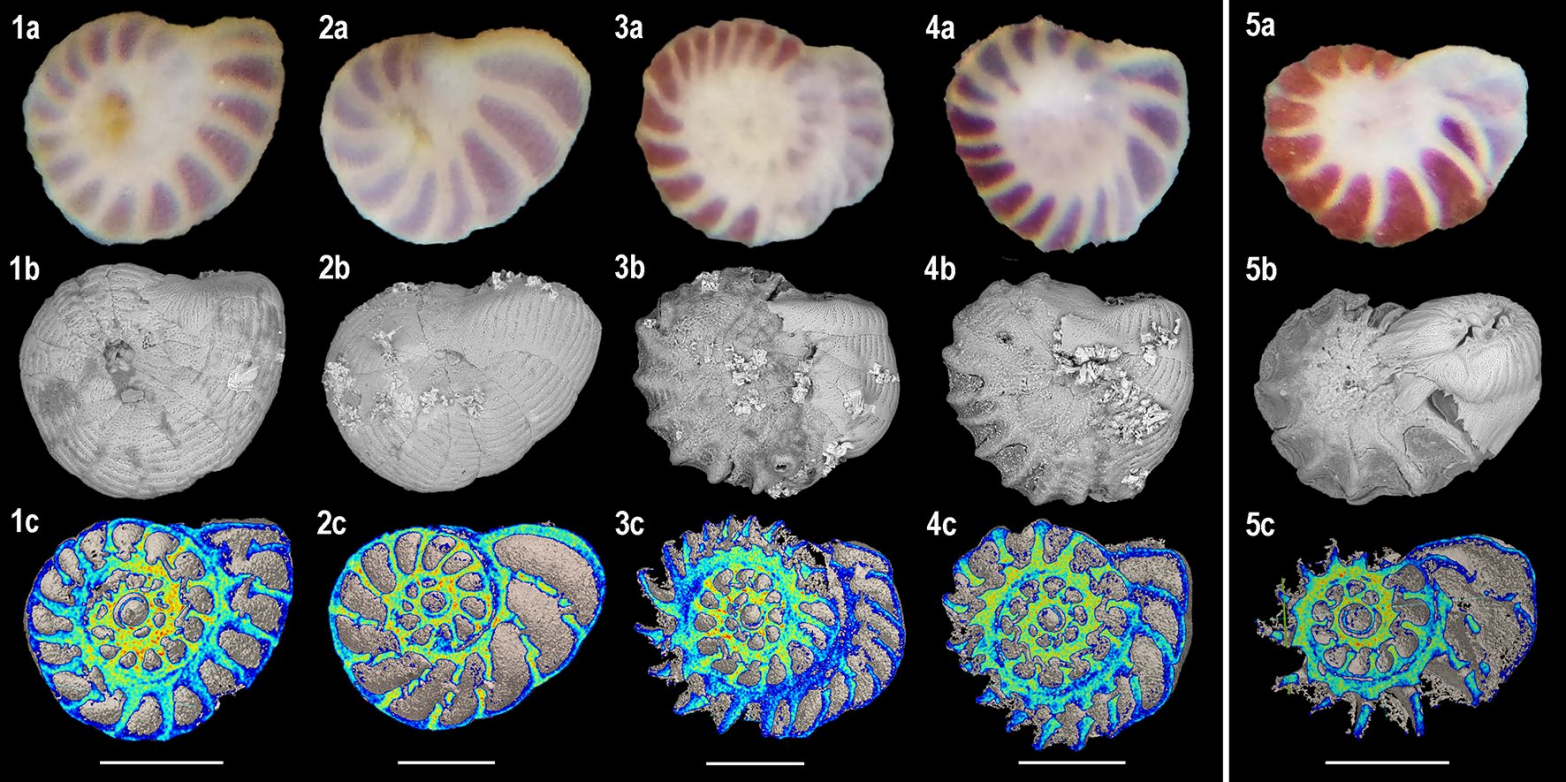
Light micrographs (above), SEM micrographs (middle) and cross-sections (below) of *Peneroplis* spp. specimens after the recovery phases. (**1**) 3 days at pH 7.9 + 32 days at pH 7.9, 1 new chamber; (**2**) 3 days at pH 7.4 + 32 days at pH 7.9, no new chamber; (**3**) 3 days at pH 6.9 + 32 days at pH 7.9 + food, 8 new chambers; (**4**) 3 days at pH 6.9 + 32 days at pH 7.9, 4 new chambers; (**5**) 32 days at pH 7.4 + 15 days at pH 7.9, 2 new chambers. Colors are relative densities. Scale bars are 200 µm.

After being back under reference pH 7.9, a new calcification phase was observed for a large majority of the specimens (Fig. 4; Table 2). This resumption of calcification took the shape of an addition of new chambers, similar to a regular calcification process. No chamber “reparations” were observed around the dissolved parts of the tests. In experiment 1, the specimens previously kept at reference pH 7.9 during the decalcification phase displayed on average 1 new chamber per specimen after the 1 month of recovery phase (Table 2). Despite being strongly decalcified, the specimens previously kept at pH 6.9 displayed on average 4 (food treatment) and 2 (no food treatment) new chambers (Fig. 4), and some specimens from the food treatment built up to 7 and 8 new chambers (Table 2). By contrast, no new chambers were observed on specimens previously kept at pH 7.4, and one specimen was considered dead at the end of the recovery phase (Table 2). In experiment 2, one specimen also died, but an average of 2 new chambers was added for the other specimens after the 15 days of recovery phase (Table 2; Fig. 4). Thus, all the specimens that resumed their calcification showed two test areas regarding calcite composition: (1) the calcite (chambers) that went through the decalcification phases and presenting varying decalcification features as described before (further called the “previous calcite”), and (2) the calcite precipitated during the recovery phases (further called the “new calcite”). Contrarily to the previous calcite, the new calcite displayed a pristine extrados layer with typical aligned rod-shaped crystals, which covers the whole surface of the newly formed chambers (Fig. 2: 4b). Despite having a pristine calcite structure, the new chambers often had abnormal size, orientation, or shape, which sometimes resulted in an altered general structure of the tests (Fig. 4: 3c, 4c, 5c). In one extreme case, a specimen showed an extra aperture after the recovery phase, which was located at the opposite side of the usual one (Fig. 2: 4a).

**Table 2:** Number of chambers added by *Peneroplis* spp. specimens during the recovery phase for both experiments. ^a^Specimens that died during that phase.

pH (time)	Experiment 1				Experiment 2
	7.9 (3 days) + 7.9 (32 days)	7.4 (3 days) + 7.9 (32 days)	6.9 (3 days) + 7.9 (32 days) + food	6.9 (3 days) + 7.9 (32 days)	7.4 (32 days) + 7.9 (15 days)
Specimen 1	1	0	8	4	3
Specimen 2	1	0	7	3	2
Specimen 3	1	0	4	2	2
Specimen 4	1	0	3	2	2
Specimen 5	0	0	2	0	0
Specimen 6	0	0^a^	1	0	0
Specimen 7	–	–	–	–	0^a^
Average	1	0	4	2	2

### Trace elements

Due to sample availability, trace elements were only analyzed on specimens from the experiment 1. Moreover, the results report only on F and F-1 chambers of specimens that built new calcite during the recovery phase (Table 2).

**Figure 5:**
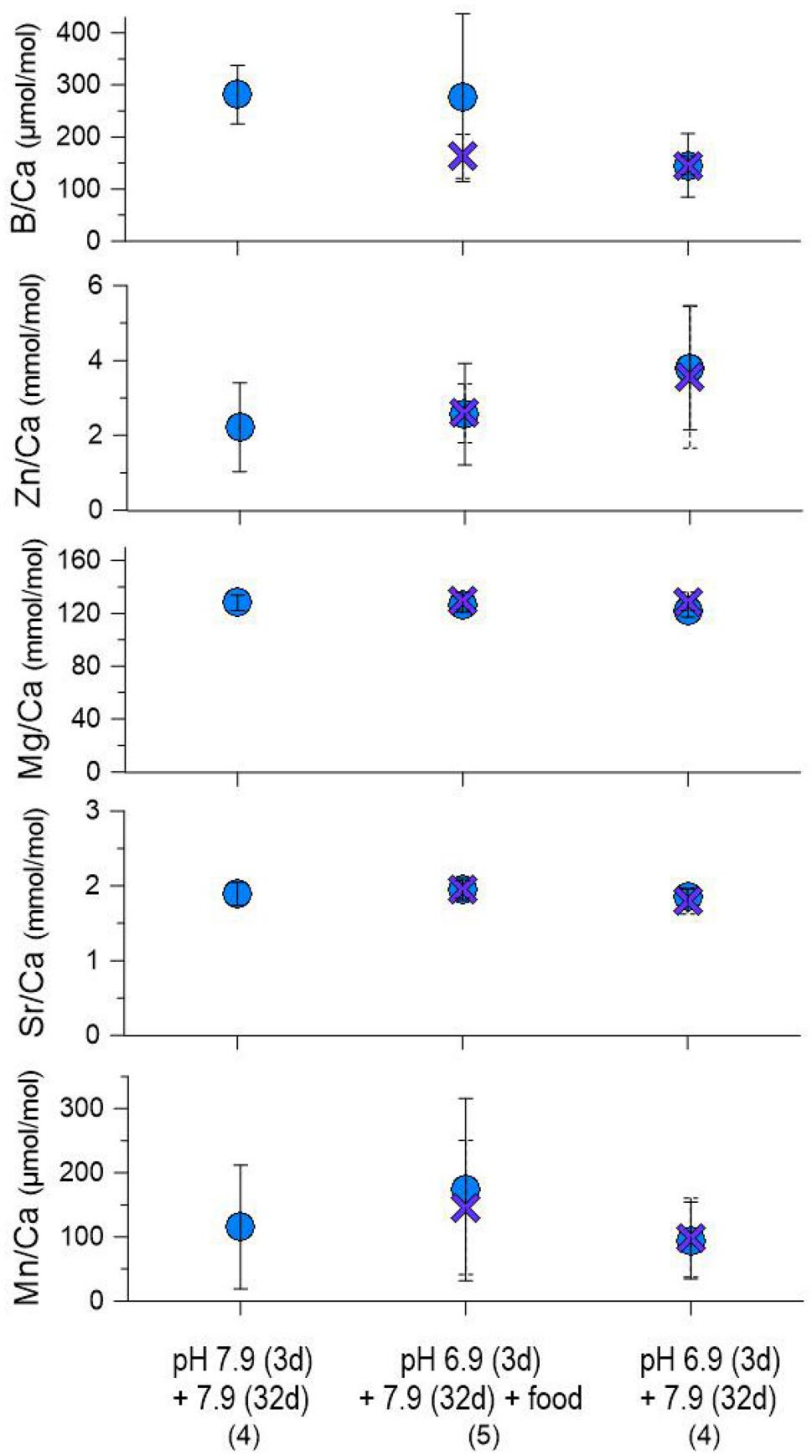
Average trace elements ratios measured on the F (blue circles) and F-1 (purple crosses) *Peneroplis* spp. chambers precipitated during the recovery phase of experiment 1. The number of specimens is indicated for each decalcification treatment. Note that the specimens from the reference pH 7.9 conditions built only one new chamber during the recovery phase, while an average of 4 and 2 chambers were built for the decalcification treatments pH 6.9 + food and pH 6.9 without food, respectively.

Average B/Ca ratios in the new calcite of the specimens previously kept at pH 6.9 during the decalcification phase and without food treatment appeared twice smaller compared to values measured on the specimens from the reference pH 7.9 treatment, with 145.5 ± 39.3 versus 281.3 ± 56.1 μmol/mol, respectively (Fig. 5). A similar but reversed trend was observed for Zn/Ca ratios, with the lowest average values of 2.2 ± 1.2 observed on specimens from the reference treatment, while an increase to 3.7 ± 1.8 mmol/mol could be observed on specimens from the pH 6.9 and without food treatment (Fig. 5).

On the contrary, the average Mg/Ca ratios on the specimens from the reference pH 7.9 treatment compared to the ones from the pH 6.9 treatments were 127.9 ± 5.8 and 126.7 ± 6.1 mmol/mol, respectively, with no clear distinction between the two food treatments (Fig. 5). Similarly, the Sr/Ca ratios in the new calcite between the reference and lower pH treatments show no visible differences, with an average value of 1.9 ± 0.1 mmol/mol for all the specimens. Including all treatments, the average Mn/Ca ratios in the newly formed chambers varied from 94.5 ± 59.9 to 173.8 ± 142.2 μmol/mol (Fig. 5).

## Discussion

High survival rates of LBF to short term OA conditions were previously observed in laboratory experiments, on specimens kept for several weeks under pH values between 8.2 and 7.6^31,33,37^. In a culture study implemented in the field, LBF specimens were able to survive at least 5 days near an hydrothermal vent where the pH was fluctuating between 7.4 and 5.9, combined with high temperature (around 40 °C^42^). Shores around Okinawa where our specimens were collected are dynamic environments with high pH variations, and the adaptation of *Peneroplis* spp. specimens to their in situ conditions is probably involved in their survival abilities under OA conditions^7^. Moreover, in all these previous experiments, day/night cycles were implemented. The symbionts’ photosynthesis provides the specimens a significant amount of energy that is supporting their regular metabolic activities^14^, and they thus help the LBF to cope with stressful environments. In our experiments, all the *Peneroplis* spp. specimens were considered alive after up to 1 month under pH 7.4, and after 3 days under pH 6.9, in both cases under dark conditions and without food addition. Our observations confirm that LBF species are able to survive low pH values on the short term, even with limited energetic resources.

Even though the seawater was saturated with respect to calcite during the pH 7.4 treatment of experiment 1 (Table 1), after 3 days test densities of *Peneroplis* spp. were lower compared to after the reference conditions treatment (pH 7.9; Fig. 3), which we linked to calcite loss. Moreover, zones with peeled extrados layer were observed at high magnification (Fig. 2). Lower test densities and dissolution zones were previously reported on LBF cultured under OA conditions, and attributed to the above 1 yet decreasing Ω_cal_ values^34,36,37^. On the species *Amphistegina gibbosa,* the observed dissolution zones were small and patchy^34^. This distribution was attributed to the ectoplasmic membrane covering the test of hyaline species that may partially protect the test from changes in the water chemistry^30,56^. In porcelaneous species this layer is absent, thus they might be more prone to test dissolution than hyaline species when cultured in the same conditions. In another study however, test densities were significantly lower for the hyaline species *Amphistegina lessonii* than for the porcelaneous species *Marginopora vertebralis* after exposure to low pH (Ω_cal_ > 1^37^). As suggested by the authors, LBF species that have adapted to a certain pH range in situ seem unable to maintain their test integrity when exposed to acidic conditions, even in saturated conditions, as a result of an imbalance in energy investment. However, as stated in McIntyre-Wressnig et al.^34^, partial dissolution and lower test density appear as non-lethal effects of short term OA conditions on LBF species.

Interestingly, calcite was still present on all our specimens after 3 days under pH 6.9, despite Ω_cal_ being below 1 (Table 1; Fig. 1: 3a, b, c). Several hyaline SBF were previously observed to survive in undersaturated conditions in culture experiments, sometimes with no signs of dissolution^57–59^. It has been demonstrated that some SBF have a tight control of their external and internal pH during the calcification process^18–20^. This ability may allow them to compensate for low pH and low Ω_cal_ and thus maintain their tests for a certain period of time that appears to be species-specific^59^. On our *Peneroplis* spp. specimens, calcite was only present in small amounts after 3 days, which is supporting these previous findings.

Severe test dissolution with large parts of the test missing was observed on our specimens after 1 month under pH 7.4, in dark conditions. Similar strongly decalcified specimens exhibiting internal organic laying were reported on SBF kept under the combined effect of low pH and very low salinity^52,59,60^. In these experiments, most of the specimens were considered alive despite the partial to complete dissolution, as attested by feeding signs and pseudopodial activity. Living decalcified specimens were also observed on the field on SBF coastal species from temperate environments worldwide (Golfe of Mexico^61^; Arcachon Bay^62^; Baltic Sea^49,53^), and attributed to acidic conditions combined with other natural and/or anthropogenic stressors. In these studies however, the dissolution was linked to great decreases in general abundances^49,61,62^, which suggests limited survival and reproduction rates of the decalcified specimens. Therefore, if our observations suggest that a heavily decalcified test does not seem necessarily lethal at an individual level, it could still negatively impact the populations at a community level.

A new phase of calcification happened for most of our specimens when put back under in situ pH values (Table 2), which highlights the recovery abilities of *Peneroplis* spp. to OA conditions. Re-calcification events were previously observed on SBF, in culture experiments (*Ammonia beccari*^51^; *Rosalina leei*^52^) and on the field (e.g. *Ammonia aomoriensis*^53^). In these studies, the calcification’s resumption was described as a repair of the dissolved parts of the test. In our case, such regeneration was not observed, and instead the resuming calcification was solely an addition of new chambers. The calcification pathway of porcelaneous species such as *Peneroplis* spp. could explain this discrepancy. Indeed, unlike the hyaline species that cover their entire test with a new layer of calcite during the calcification process, porcelaneous species have non-lamellar walls which they are unable to repair following damage^56^. This addition of new chambers was remarkable and demonstrates the ability of *Peneroplis* spp. to absorb damages from short-term pH decrease.

Slightly dissolved specimens previously kept 3 days under pH 7.4 did not show calcification recovery features after 32 days back under in situ pH, whereas most of the individuals from the reference condition built on average 1 new chamber (Table 2). The short period of lower pH may have been a source of mild stress for the specimens, which from then might have relocated their energetic resources to respiration or homeostasis rather than calcification. By contrast, the large number of new chambers built by the heavily decalcified specimens previously kept 3 days under pH 6.9 seems to be a survival reaction after highly stressful conditions. Similar observations were made in a culture study by Le Cadre et al.^51^, where the partially decalcified hyaline foraminifera slowly started to re-emit pseudopodia after being placed in the rescue solution, while the strongly decalcified specimens showed an immediate and intense pseudopodia activity as soon as back at in situ pH. In the case of our 1 month under pH 7.4 treatment, such intense survival response was not observed despite the strong decalcification of the specimens, and only 2 new chambers were added on average (Table 2). This could be explained by the combination of lack of energy and time, as the recovery phase in experiment 2 lasted only 15 days and as no food was provided (see below). Thus, our data seems to indicate a threshold level regarding test dissolution/recovery of porcelaneous species, after which a survival response in the form of precipitation of new chambers is induced as soon as the specimens are placed back under more optimal pH condition.

In our experiments, the number of chambers formed during the recovery phases was on average higher when food was provided (Table 2). This is coherent with previous culture experiments where porcelaneous species showed higher growth rates in fed conditions than in unfed conditions^63^. A better resistance to reduced pH in terms of growth and calcification rates was observed for many other calcifiers when food was provided (review in Ref.^6^). Indeed, most coping mechanisms to varying pH are metabolically demanding, and this energy supply becomes then significantly important. Our results demonstrate that food supply is a crucial parameter to integrate in culture experiments in order to accurately estimate the effects of OA conditions on foraminifera.

The newly built chambers had a pristine appearance, with a perfectly calcified extrados layer (Fig. 2: 4b). Smooth surface of the re-calcified chambers compared to previous chambers was also observed in Le Cadre et al.^51^. However, similarly to the observations by Le Cadre et al.^51^ and Kurtarkar et al.^52^, the new chambers had abnormal shape, size, or orientation, and the general structure of the tests was subsequently affected. Abnormal tests are usually attributed to stress such as pollution^64^ or variability in the environment^65^, but in cases where dissolution happened, physiological or structural damages of the test during the decalcification phases were suggested^52^. Indeed, in foraminifera, the morphology of a new chamber is linked to the morphology of the previous chambers, so if damages have occurred on the test then the next chambers will be affected^66^. This was also observed many times in our laboratory during previous experiments (unpublished data). On our specimens, which already display non-repaired chambers, such deformations of the new chambers could impair their resistance to attack from other organisms as well as to physical shocks, with further serious implications for the survival and reproduction of *Peneroplis* spp. in situ.

The B/Ca ratios (145.4 ± 17.6 to 281.3 ± 56.1 μmol/mol; Fig. 5) in our *Peneroplis* spp. were in the range of the only previously published measurement on a high-Mg calcite LBF, on the species *Amphisorus hemprichii* (500 ± 204 μmol/mol^28^). In our experiment, the stress induced by 3 days under low pH 6.9 in dark conditions combined with no food given during the recovery phase has lowered the B incorporation into these specimens, in comparison to our reference conditions where specimens were maintained under pH 7.9 during the whole length of the experiment. These results are in good agreement with previous culture studies on both LBF^67^ and planktonic foraminifera^68–70^, where B/Ca showed decreasing trends under lower pH. Our results highlight the LBF B/Ca signatures response to pH variations, and their potential use as a seawater carbonate chemistry proxy.

The Zn/Ca ratios measured in our *Peneroplis* spp. (2.2 ± 1.2 to 3.8 ± 1.7 mmol/mol; Fig. 5) were high compared to the only ones previously observed in *P. pertusus* (53 ± 10.8 µmol/mol^27^). The dark conditions during the decalcification phase in our experiment are probably involved in this offset. Indeed, Zn/Ca has been described as a nutrient proxy^71^, and Zn is known as an essential micronutrient for microalgae with a critical role in the photosynthesis process^72^. Therefore, the symbionts got probably stressed by the dark period, affecting the Zn incorporation in all our specimens. Furthermore, our results are coherent with this previous study on *P. pertusus*, where Zn/Ca ratios were also increasing under lower pH^27^.

The Mg/Ca ratios in *Peneroplis* spp. from our study (122.4 ± 5.3 to 129.4 ± 6.7 mmol/mol) were similar to the ones in *Peneroplis planatus* (90 to 175 mmol/mol^73^), and in *Peneroplis pertusus* (126.1 ± 1.8 mmol/mol^27^), while our Sr/Ca ratios (1.8 ± 0.2 to 2.0 ± 0.1 mmol/mol) were also comparable to previously reported values for *P. pertusus* (2.1 ± 0.07 mmol/mol^27^). Despite the large error bars, our Mn/Ca values (94.5 ± 59.9 to 173.8 ± 142.2 μmol/mol; Fig. 5) were in the range of concentrations observed in the high-Mg calcite LBF *Sorites marginalis* (61.7 ± 27.4 μmol/mol^74^). In previous culture experiments, these three E/Ca ratios have been correlated with diverse carbonate system parameters^36,57,74,75^. However, in our study, the 3 days under low pH 6.9 during the decalcification phase had no visible impact on the Mg, Sr, and to a lesser extent Mn incorporation in *Peneroplis* spp. calcite during the recovery phase.

Our results suggest that the incorporation of elements such as B and Zn is rapidly impacted by a change in the environment, and only three days at lower pH and dark conditions was sufficient to modify the geochemical signature in *Peneroplis* spp. when the calcification restarts. Even though LBF E/Ca appear promising as environmental changes proxies, multi-elemental approaches and species-specific calibrations are still necessary to reinforce their reliability.

## Materials and methods

### Sampling

Surface sediment containing live specimens of *Peneroplis* spp. was collected by hand on shores along Ikeijima Island, Okinawa, Southwest Japan (26° 23′ N, 128° 00′ E), in May 2019. Sediment was transported to JAMSTEC (Japanese Agency for Marine-Earth Science and Technology, Japan), and stored in its original seawater at in situ temperature (T, 25 °C) under a 12 h day/night cycle, for one month of acclimation. During this period, salinity and pH (Total Scale) were monitored every week in the aquarium, and were on average 36.33 ± 1.49 and 7.9 ± 0.06, respectively. This pH value was used as reference value in our experiments. Scarce genetic analyses were found in the literature for the *Peneroplis* genus (e.g. Ref.^76^), and due to the natural polymorphism observed in *Peneroplis* spp. populations, morphological identification to the species level remains unclear^77,78^. In this study, we use a conservative approach and refer to our specimens as *Peneroplis* spp. Reproduction happened several times in the aquarium as attested by the presence of juveniles. Adult individuals (showing at least 10 chambers) from various generations issued of reproduction within the aquarium were randomly chosen for culture experiments. The living status of selected specimens was established by direct observation of cytoplasm streaming and pseudopodia emission with a Zeiss Axio Observer Z1 inverted microscope.

### Culture experiments

We conducted two culture experiments that are comprise both of two phases: a low pH conditions phase (called the decalcification phase), followed by a reference pH conditions phase (called the recovery phase). To avoid potential pH effects of photosynthesis by the symbionts, the decalcification phases happened in the dark. By contrast, a day/night cycle was applied during the recovery phases. A flowing system was used to ensure that the intended pH values were reaching the foraminifera microenvironment in a realistic way, as recommended for OA studies^16,17^. The experiments took place in an incubator that was kept constant at 25 °C during both phases.

In experiment 1, three different pH treatments were tested: 7.9, 7.4 and 6.9. We altered the pH in the three tanks by bubbling pure CO_2_, in a circulating water system similar to the one described in Dissard et al.^57,79^, in Schiebel and Hemleben^80^ and adapted as described in Charrieau et al.^59^. Culture cups (3 mL) containing 9 living specimens each were placed in the system: culture cup A in tank 1 (pH 7.9, reference conditions), culture cup B in tank 2 (pH 7.4), and culture cups C and D in tank 3 (pH 6.9). The decalcification phase lasted 3 days. Light micrographs of each specimen were taken every day to observe survival and test dissolution features. At the end of the decalcification phase, 2 or 3 specimens from each culture cup were removed for Scanning Electron Microscope (SEM) imaging and Micro X-ray Computed Tomography (MXCT) scanning, and one specimen per condition was further selected for Environmental SEM (E-SEM) imaging. All the culture cups were then placed in reference conditions (tank 1, pH 7.9) for the recovery phase, which lasted 32 days. During this period, LBF from culture cup C were fed twice a week with *Thalassionema* sp. Light micrographs of all the specimens from the four culture cups were taken twice a week to observe survival and calcification recovery features. At the end of the experiment, SEM imaging and MXCT scanning were performed on all the specimens. Additionally, one specimen per condition was selected for E-SEM imaging, and five specimens per culture cup were selected for trace elements analysis of their tests.

In experiment 2, one culture cup E containing 12 live specimens was maintained under pH 7.4 (tank 2) for the decalcification phase, which this time lasted 32 days. At the end of the decalcification phase, one individual was removed for SEM imaging and MXCT scanning. Then, the culture cup was moved to reference conditions (tank 1, pH 7.9) for the recovery phase, which lasted 15 days. Food was not given during the second experiment. Light micrographs were taken twice a week during both phases to observe survival and decalcification/calcification recovery features. At the end of the experiment, SEM imaging and MXCT scanning were performed on all specimens and one specimen was selected for E-SEM imaging.

### Seawater carbonate chemistry

Salinity, pH (Total Scale) and alkalinity (ALK) within the tanks were measured at the beginning, middle, and end of both experiments. ALK and pH were determined by the one point titration method described in Dickson et al.^81,82^, but simplified for small volumes according to in-house standards^83^. The water pH and HCl titrated water pH were measured with an ion meter (ORION 4 STAR, Thermo SCIENTIFIC) set with a micro composite electrode (PerpHecT^®^ ROSS^®^ Micro Combination pH Electrode, Thermo SCIENTIFIC). The CO_2_calc software program^84^ was used to estimate other carbonate system parameters such as calcite saturation state (Ω_cal_), [CO_3_^2−^] and [Ca^2+^] (Table 1). ALK, pH, S, and T were used as inputs parameters. Equilibrium constants from Lueker et al.^85^ were applied for K1 and K2, and total boron ratio from Uppström^86^ was used, as recommended by Orr et al.^87^.

### SEM and E-SEM imaging

The foraminifera selected for Scanning Electron Microscope (SEM) imaging were left to dry at room temperature, mounted on aluminum stubs, and placed in a Hitachi Miniscope TM3000, JAMSTEC.

Selected specimens for Environmental SEM (E-SEM) imaging were left to dry at room temperature, mounted on an aluminum stub, and covered with a thin layer of osmium coating, prior to be positioned in a Quanta 450 FEI, JAMSTEC.

Test dissolution and calcification recovery features were assessed visually by observation of the resulting images, and the specimens’ images from different pH treatments were compared.

### MXCT scan

Selected specimens for Micro X-ray Computed Tomography (MXCT) scanning were mounted on stubs and analyzed with an X-ray scanner, JAMSTEC (X-ray voltage = 80 kV, tube current = 40 µA, target current = 10 µA, detector matrix = 1024 × 1024 pixels, 1800 projections, spatial resolution = 1.00 µm/voxel). A grain of calcite crystal (NIST SRM8544, Limestone, NBS 19) was scanned together with each specimen as a reference standard material. We used an aluminum filter (thickness = 0.20 mm) to avoid the beam hardening effect. The Molcer-Plus software (White-Rabbit Inc, Japan) was used to reconstruct 3D and cross-section images. The CT number for each specimen was calculated and normalized based on the calcite standard values (NBS 19; 2.71 μg/μm^3^ in density; 1000 in mean CT number), using the Eq. (1):

1 $${\text{CT}}\,{\text{number}}_{{{\text{sample}}}} = \, \left( { \, \mu_{{{\text{sample}}}} - \, \mu_{{{\text{air}}}} } \right) \, / \, \left( { \, \mu_{{{\text{calcite}}}} - \, \mu_{{{\text{air}}}} } \right) \, \times {\text{ CT}}\,{\text{number}}_{{{\text{calcite}}}} ,$$

where μ is the X-ray attenuation coefficient (voxel number). The reference standard deviation (RSD) of the MXCT measurements was 0.84%.

As there is a direct relation between the CT number and the shell bulk density, we calculated the shell bulk density for each specimen using the regression Eq. (2), based on artificial calcite material with known densities:

2 $${\text{Bulk}}\,{\text{density }}(\upmu {\text{g}}/\upmu {\text{m}}^{{3}} ) \, = \, \left( {{\text{CT}}\,{\text{number }} - { 49}.0{4}} \right) \, /{ 35}0.{47} \left( {{\text{R}}^{{2}} = 0.{999}} \right).$$

### Laser ablation-ICP-MS

Foraminifera from culture experiments, alive at the time of collection, do not require the rigorous cleaning procedure applied to dead/fossil specimens. Instead, the modified cleaning procedure described in Dissard et al.^57,79^ and Fontanier et al.^88^ was adopted. Organic matter was removed by soaking specimens for ~ 35 min in a 3–7% NaOCl solution prior analysis^89^. Directly after complete bleaching, the specimens were thoroughly rinsed with deionized water to ensure complete removal of reagents. The last two chambers (F and F-1) of *Peneroplis* spp. specimens were analyzed (see Supplementary Fig. S1). The chambers were ablated using an Nd–YAG Laser (NWR-213, New Wave, ESI) inside an ablation chamber flushed with Helium (0.7 L/min), at the ALYSES Analytical Platform (Centre IRD France Nord). The ablated sample was then mixed with an Argon flow (0.6 L/min) and transported to an Inductively Coupled Plasma Mass Spectrometry (ICP-MS). Pulse repetition rate was set at 10 Hz, with an energy density at the sample surface of 30 J/cm. Ablation craters were 40 μm in diameter and ablated material was analyzed with respect to time (and hence depth) using an ICP-MS (7500cx Agilent). Two measurements were performed per chamber. Analyses were calibrated against NIST SRM 610 glass, using the concentration data of Jochum et al.^90^ with ^43^Ca as an internal standard. Concentrations of Mg, B, Mn, Zn, and Sr were calculated using ^24^Mg, ^11^B, ^55^Mn, ^66^Zn, and ^88^Sr. An in-house matrix matched carbonate standard was used to verify potentially different ablation behaviour.

## Supplementary Information

Supplementary Figure S1.
