# Supplementary material for: The coral reef-dwelling Peneroplis spp. shows calcification recovery to ocean acidification conditions
Source: Sci Rep. 2022 Apr 16;12:6373. doi: 10.1038/s41598-022-10375-w (PMC9013382; doi:10.1038/s41598-022-10375-w)
Supplement: Supplementary file 1 — Supplementary Figure S1. [file 41598_2022_10375_MOESM1_ESM.pdf]

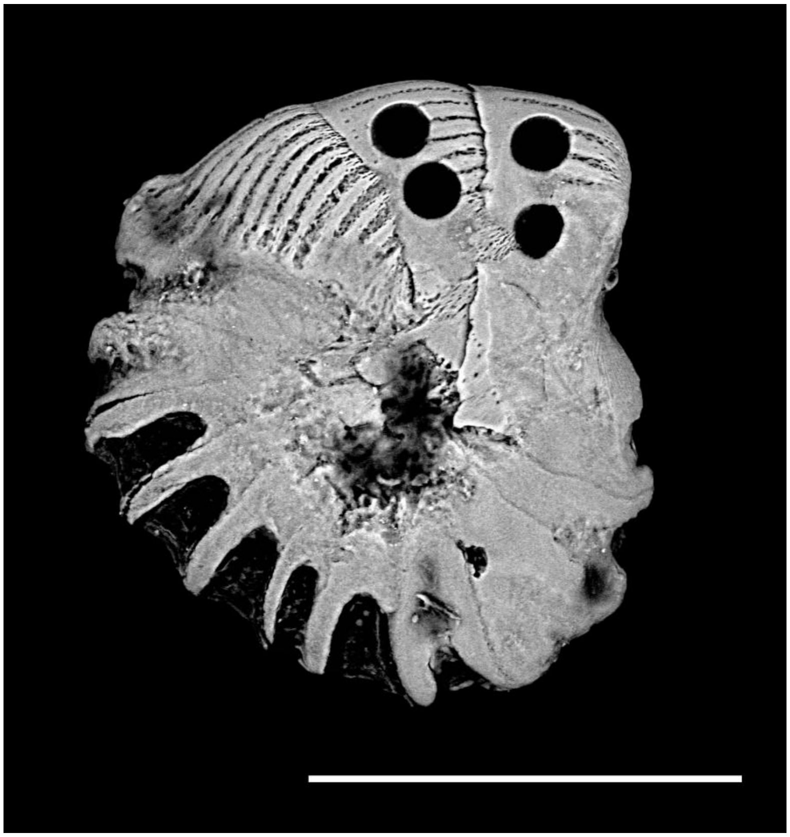

Figure S1. SEM micrograph of laser-drilled spots on the last two chambers (F and F-1) of *Peneroplis* spp. test during LA-ICP-MS measurement. Scale bar: 300 µm.
